# Supplementary material for: Are carbon emissions trading and green financial instruments synergistic? -Comprehensive quantitative research based on content analysis
Source: PLoS One. 2024 Mar 7;19(3):e0298601. doi: 10.1371/journal.pone.0298601 (PMC10919601; doi:10.1371/journal.pone.0298601)
Supplement: S1 Data — (ZIP) [file pone.0298601.s001.zip › Data/地级市Z_F工作报告.zip!/╔╜╬≈/╜·╓╨╩╨/└┤╫╘╣1⁄2╓┌║┼í╛┬φ┐╦╩2╛▌═°í┐_2016.docx]

各位代表:

现在,我代表市人民政府向大会作报告,请予审议,并请政协委员和其他列席人员提出意见。

一、本届政府工作回顾

市三届人大一次会议以来,面对严峻复杂的经济形势和艰巨繁重的改革发展任务,我们在省委、省政府和市委的坚强领导下,在市人大、市政协的有力监督和大力支持下,围绕率先发展、富民强市总目标,抢机遇、抓关键、建机制、补短板、破难题,经济社会发展取得新成就,为“十三五”全面建成小康社会打下了坚实基础。

第一,经济实力快速提升。全力推进项目建设提质增效加速,持续增加有效投资,共完成项目投资5304亿元,带动全社会固定资产投资年均增长24.3%,从2015年4月开始,全市固定资产投资增幅连续17个月领跑全省。因地制宜创造供给、引导消费,社会消费品零售总额由2009年的234.2亿元增加到529.7亿元,年均增长13.9%。积极实施企业帮扶行动,大力促进实体经济发展,有效保持经济稳定运行。全市地区生产总值由2009年636.6亿元增加到2015年1046.1亿元,突破千亿元大关,年均增长10%;一般公共预算收入由57.8亿元增加到100.2亿元,年均增长9.6 %,总量跃升至全省第二;规模以上工业增加值由273.5亿元增加到379.5亿元,年均增长13.6%,综合实力迈上了新台阶。

第二,产业转型步伐加快。坚持煤电一体化、装备高端化、农业现代化、旅游集团化、业态新型化,推动产业结构优化升级,全市三次产业结构由2009年的8.7:51.9:39.4调整为2015年的10.1:43.8:46.1,第三产业比重增加6.7个百分点。改造提升煤焦产业,全面完成煤炭资源整合、煤矿兼并重组,单井产能由29.81万吨/年提高到92.74万吨/年;落地低热值煤发电项目5个。发展高端装备制造业,建成吉利新能源汽车、太重榆液等一批新型装备制造项目。全面实施国家现代农业示范区,推进现代农业经营模式创新,粮食连续6年获得丰收,达到17.58亿公斤,比2009年增长27%;肉蛋奶总产实现翻番,蔬菜产量突破50亿公斤,稳居全省前列;全市农业龙头企业加工流通销售收入比2009年增长211%。大力推动文化旅游融合发展,全市旅游接待游客量和总收入年均增幅分别达到34%和39%,去年旅游总收入突破500亿元,跻身中部旅游城市第一方阵,跨入全国旅游城市六十强,文旅融合发展经验在全省推广。加强文化晋中建设,促进文化产业多元化发展,文化产业增加值和占GDP比重保持全省领先。加快培育休闲度假、健康养老、居家服务等新兴业态,引进社会资本实施了晋中万达广场、天美杉杉奥特莱斯、百草坡周末度假村、三晋主题乐园、潇河水上乐园等一批重点项目,中鼎物流园区全面开工,服务业对GDP增长贡献率达47.7%,成为经济增长新引擎。

第三,城乡面貌焕然一新。加快市城区扩容提质,连续5年实施“百亿市政重点工程”,新建规划馆、博物馆、图书馆、科技馆、工人文化宫等一批标志性项目;完成道路快速化改造30.99 公里,新增公交专用车道37.8公里,晋中太原城际铁路2号线开工,城区公共自行车首批5000辆投用;新增公园绿地面积878万平方米,是2009年前绿地面积总和的2倍多;城市配套设施进一步完善,污水处理率、燃气和集中供热普及率分别达96.81%、96.71%、98.49%;城市建设管理水平迈上新台阶,跻身省级文明城市行列。大力改善市域交通条件,新增公路通车里程1592公里,公路密度百平方公里比2009年增加近10公里,基本形成“六纵六横六循环”公路网骨架和一小时交通圈;龙城、汾邢、阳黎3条高速公路建成通车,实现高速公路“县县通”;新建改建国省干线公路 100.4公里、农村公路2501 公里,400人以上自然村全部通水泥油路,行政村通客车率达91.2%。积极推进水利建设,松塔、恋思、石膏山、口上4座新水源工程开始供水,东山供水、中部引黄两大水网工程顺利推进,新增供水能力1.31亿立方米,61.7万人的饮水安全质量得到改善提高,农村自来水普及率达95%。不断完善电力设施,全面完成新一轮农网改造升级工程,新增35千伏及以上变电容量3481兆伏安,解决了8.6万户群众“低电压”问题。

第四,生态文明建设成效明显。强力推进节能降耗,万元GDP综合能耗下降17.32%,化学需氧量、氨氮、二氧化硫、氮氧化物削减率分别为15.76%、18.64%、11.3%、23.88%,万元工业增加值用水量降低27.4%,全部提前超额完成“十二五”目标任务。2015年市城区空气二级以上天数251天,优良率68.8%,空气质量综合指数6.63。持续开展造林绿化,全市林木绿化率年均增长1个百分点,达到35.83%,6个县(区、市)达到省级林业生态县标准;省级以上森林公园达到22个、湿地公园7个,所有县城周边全部拥有生态公园。

第五,改革开放不断深化。转型综改扎实推进,晋中108廊带区域一体化发展示范区列入省级战略并全面启动建设,总投资110亿元的10大系统工程全面开工,完成投资55亿元。农村改革全面落实,完成县级农村产权流转交易中心建设,组建现代农业融资担保公司,在全省首开果树政策性保险试点,农业保险范围覆盖粮食、畜牧、蔬菜、林果产业。

金融领域改革成效明显,天交所山西运营中心在我市落户,瑞阳供热首开全省资产证券化融资先河,凯嘉集团全省率先发行民营企业债券,平遥日升隆首发全省第一单小贷公司私募债,新增贷款增幅全省第一。推进政府和社会资本合作,储备和实施总投资450亿元PPP项目48个,社会投资占到九成以上。土地管理不断加强,在全省首家出台地下空间建设用地出让办法,依法无偿收回闲置土地1442.7亩,累计盘活闲置低效土地3.87万亩,占供地总量30.7%。科技创新能力稳步提升,建成一批国、省级技术研发中心和科技孵化器,“双创基地”、晋中众创家园投入运行。对外开放和招商引资成果丰硕,累计引进省外投资1642亿元。

第六,民生保障持续改善。全面实施就业创业工程,6年城镇新增就业25.79万人,转移农村劳动力29.94万人,城乡居民人均可支配收入分别达到27525元和10877元,是2009年的1.88倍和2.09倍。切实加强社会保障,完善企业养老保险基金统收统支管理办法,城乡居民基本养老保险制度实现“并轨”,各项社会保险扩面征缴任务全面完成,全国养老服务业综合改革试点工作取得积极进展。优先发展教育事业,在全省率先通过县域义务教育基本均衡国家评估认定,教育综合改革成为全国典型,山西高校新校区落地我市,10所院校12万学生入驻。扎实推进医药卫生事业改革发展,县级公立医院改革全面完成,在全省率先开展乡村医疗卫生“五统一管理”,幸福家庭创建成为全国首批、全省唯一示范城市,卫生计生融合发展走在全省前列。大力发展文化事业,全面实施全民文化行动,文艺精品创作成果丰硕,公共文化服务体系建设跨入全国先进行列。

加快发展体育事业,大力实施全民健身活动,率先在全省完成了农民健身全覆盖工程。扎实推进扶贫开发,实施易地搬迁、片区开发、整村推进等重点工程,完成16.69 万贫困人口脱贫。全面落实保障性安居工程建设任务,开工建设各类保障性住房13.37万套,解决了5.36万户城镇家庭和2.69万户农村家庭的住房困难。狠抓安全生产工作,严格落实安全生产责任,实施分类分级动态管理和“清单化监管”,推行工商注册安全监管全覆盖,各类安全生产事故起数和死亡人数比2009年分别下降57.42%和⦃65.32%⦄,安全生产形势稳定好转。深化平安晋中建设,构建立体化社会治安防控体系,推进矛盾纠纷“大调解”机制建设,推行基层社会治理“网格化”服务,社会治安综合治理工作走在全省前列。

第七,政府建设全面加强。自觉接受人大法律监督和政协民主监督,严格执行人大及其常委会的决议、决定,积极支持人民政协履行职能,市政府各部门共办理人大代表意见、建议和议案1236件,政协委员提案1937件,满意率达到99%以上。加强效能政府建设,积极推进依法行政,在全省率先公布涉企权力清单,推行“两集中、两到位”行政审批制度改革,实行行政处罚权相对集中管理;推行公共资源市场化配置,在全省率先公开出让天然气加气站、停车场、广告位等经营权,商业和地产开发项目变传统招拍挂为设定公开商业条件、招商招标合并竞争;引进第三方评估,推行政府购买服务和部门绩效管理,健全公共权力接受社会监督常态化机制。严格落实党风廉政建设主体责任和“一岗双责”,狠刹“四风”,严惩腐败,各级干部的责任意识、服务意识、廉洁从政意识进一步增强,干事创业的氛围日益浓厚。

同时,粮食供销、外事侨务、文物保护、防震减灾、民族宗教、档案史志、应急管理、对台、妇女儿童、老龄、红十字、残疾人、气象等工作取得了新成绩。国防动员、人民防空、双拥共建、民兵预备役建设进一步加强。

过去的六年,是我市发展史上综合实力提升最快、城乡面貌变化最大、人民群众得到实惠最多的时期之一。这里,我郑重向大会报告,市三届人大一次会议提出的目标任务全部完成!“十二五”规划的主要任务圆满完成!市人民政府历年向全市人民承诺的实事全部兑现!

各位代表,晋中的变化有目共睹,发展的成就令人欣慰。这些成绩的取得,是省委、省政府和市委坚强领导、科学决策的结果,是市人大、市政协大力支持、有效监督的结果,是全市广大干部群众苦干实干、团结奋斗的结果。在此,我代表市人民政府,向全市人民,向所有关心支持晋中发展的各界人士,表示衷心的感谢和崇高的敬意!

成绩来之不易,经验尤为可贵。六年来的实践不仅取得了丰硕的发展成果,也带给我们许多有益的启示,最重要的就是:必须始终坚持发展第一要务,把加快发展作为解决一切问题的关键,充分发挥自身优势,坚定不移抓项目、稳增长、促转型,推动经济提质增效,加快赶超发展步伐。必须始终坚持改革创新,尊重客观规律,致力破解制约和阻碍发展的体制机制障碍,不断激发加快发展的内生动力,释放企业和社会创新活力。必须始终坚持保障和改善民生,着力解决人民群众最关心、最直接、最现实的利益问题,使发展成果更多更公平地惠及全市人民。必须始终坚持依法行政,尊崇法治、敬畏法律,自觉运用法治思维和法治方式开展工作,不断提高政府的公信力和执行力。必须始终坚持担当负责,

保持奋发有为、昂扬向上的精神状态,真抓实干,攻坚克难。

在肯定成绩的同时,我们也清醒地看到存在的问题,主要是:经济总量不大,质量效益不高,传统产业不强,新兴产业偏弱,中小企业发展活力不足;发展方式比较粗放,自主创新能力不强,节能减排和环境保护还需加大力度;城乡区域发展不平衡,城镇化水平不高,社会事业发展欠账较多,就业、社会保障工作压力较大,农民增收特别是贫困地区全面脱贫任务艰巨;思想解放不够,政府职能转变、干部作风建设有待加强,发展环境还需进一步改善。对此,我们必须高度重视,切实加以解决。

二、下届政府工作总体要求和主要任务

当前,面对经济发展进入速度变化、结构优化、动力转换的新常态,面对全省各市竞相发展的新形势,落实省委构建良好政治生态、推动经济稳步向好的决策部署,实施“十三五”规划,实现市第四次党代会确定的目标任务,我们必须充分发挥自身独特的优势。这些优势主要体现在:山西中部城市群的规划实施,太原晋中一体化的加速推进,以及山西高校新校区、山西科技创新城、山西新能源汽车园区、中鼎物流园区的全面建设,将使晋中独特的区位优势更加凸显,形成要素加速集聚的新优势。我市资源禀赋得天独厚,产业基础扎实,纺机、液压元件、玻璃器皿、玛钢铸造以及现代农业、文化旅游等特色产业加速壮大,承接东部省份加工贸易产业转移条件优越,将在产业转型升级大潮中赢得先机,形成经济多元增长的新优势。我市被确定为国家级现代农业示范区、全国创新驱动示范市以及全国中小城市综合改革、全国综合行政执法体制改革等试点,108廊带区域一体化示范区上升为省级战略,今年又成为20个受到国务院通报表扬的地级市之一,将在改革创新方面获得更大空间,形成发展动能加快转换的新优势。我市近年来投资环境不断优化,政府职能转变和管理创新步伐加快,各级干部干事创业的信心和劲头更加充足,将为经济社会发展提供更加有力的保障,形成政府效能提升的新优势。我们一定要树立强烈的机遇意识、责任意识、创新意识和发展意识,理直气壮抓发展,聚精会神抓经济,积极应对挑战,奋力攻坚克难,把拥有的诸多优势加速转化为新的发展优势,全力谱写晋中改革发展新篇章!

做好新一届政府工作的总体要求是:高举中国特色社会主义伟大旗帜,深入贯彻习近平总书记系列重要讲话精神,按照省委“一个指引、两手硬”的重大思路和要求,贯彻市第四次党代会精神,以发展为第一要务,以改革为根本动力,以创新驱动、转型升级为主基调,培育厚植比较优势,实施转型带动、项目拉动、改革推动、创新驱动四大战略,全力推动经济社会持续健康发展,确保如期实现全面挺进全省第一方阵、全面建成小康社会“两个全面”奋斗目标。

实现“两个全面”奋斗目标,就是要到2019年全面挺进全省第一方阵 ,到2020年与全国同步全面建成小康社会。全面挺进全省第一方阵与全面建成小康社会,是一个进程,两个阶段。全面挺进第一方阵,是全面建成小康社会必须首先打胜的“晋中战役”,就是要苦干三年,经济发展硬指标挺进全省前列,区域影响软实力走在全省前列,脱贫攻坚硬任务力争提前完成。

经济社会发展的主要任务是:

——经济实力跃上新台阶。到2019年底,体现经济总量的地区生产总值、体现经济主要支撑的规模以上工业增加值、体现拉动经济发展主要动力的固定资产投资、体现综合实力的财政收入和体现群众生活水平的城乡居民可支配收入6项指标绝对额进入全省前列。保持经济中高速增长,到2020年地区生产总值和城乡居民人均收入比2010年翻一番。

——产业升级取得新进展。传统产业改造提升焕发新的生机和活力,特色产业建成一批全国性的品牌基地,新兴产业逐步成为经济增长新的支撑,以“互联网+”为核心的新业态和新模式经济比重持续提升,产业迈向中高端水平,构建晋中现代产业体系。

——城乡统筹形成新格局。区域协调互动发展的整体性进一步增强,城市空间布局更加优化,基础设施不断完善,综合承载力和建设管理水平全面提高,城乡一体化发展格局基本形成。

——改革创新实现新突破。108廊带区域一体化发展示范区建设取得重要进展,国家现代农业示范区、国家级教育信息化试点、全国中小城市综合改革试点扎实推进,各项重点领域和关键环节改革取得决定性成果。创新集聚能力不断增强,科技、人才、创业、创新高地效应进一步显现。

——生态文明建设再上新水平。能源和水资源消耗、建设用地、碳排放总量得到有效控制,大气、水环境质量达到国家标准,完成国家和省约束性考核指标,打造资源型经济绿色转型先行区、山西中部城市群生态宜居城。

——民生社会发展取得新成效。农村贫困人口全部脱贫,四个国定、省定贫困县全部摘帽;率先基本建成现代化教育强市,就业比较充分,城乡公共服务和保障体系基本健全,文化强市建设取得明显成效,安全生产向稳定好转坚实迈进。

——民主法治建设迈出新步伐。人民民主更加健全,法治政府基本建成,司法公信力明显提高;服务型政府建设成效显著,政府公信力和行政效率进一步提高,社会治理能力和水平不断提升,社会更加和谐稳定。

围绕完成上述任务,新一届政府要重点抓好八个方面工作:

(一)持续增加有效投资,充分发挥投资对经济增长的关键作用

坚持大企业引领、大项目带动,突出抓好有效投资,确保固定资产投资保持较高增速,形成对经济增长稳定持久的支撑。

深入实施大项目攻坚。围绕省、市确定的十大重点领域,依托市场需求、优势产业链延伸、国际国内大企业大集团生产力布局,谋划实施一批重大转型项目,推动经济结构战略性调整;围绕推进城乡统筹、生态治理、民生改善和社会建设,谋划实施一批重大基础设施工程和公共服务项目,努力补齐经济社会发展短板;围绕培育文化体育、休闲消费、健康养老等新需求、新业态,谋划实施一批重大服务业项目,加快推动消费升级。坚持项目建设“六位一体”,不断创新项目推进服务机制,全力加强项目储备和签约,加快在库项目落地、投产、见效,有效支撑经济稳定增长。

全面加大招商引资力度。积极参与国家和全省开放发展战略,主动承接东部地区加工贸易产业转移,以大开放促进大招商,以大招商带动大投资、推动大发展。瞄准世界500强、国内500强,积极与中央和省属大企业结成战略联盟,找准市场需求和我市优势的结合点,抓好项目、产业链、产业集群点线面结合系统招商,提高项目招商精准度、成熟度。统筹布局招商引资项目,坚持全市一盘棋,大力运用“飞地经济”等模式,推动招商引资项目落地,实现资源利用效益最大化、企业投资成本最小化、各地利益分享最优化。“十三五”期间,全市招商引资签约项目总投资达到1万亿元以上。

全力激活民间投资。积极创优民间投资环境,建立市场准入负面清单制度,坚决清除各类制约障碍,降低制度性交易成本,提振民间投资信心。大力推进PPP项目储备和实施,完善政府和企业风险分担、互惠共赢的合作机制,鼓励社会资本参与重大项目和重点工程建设。有效破解民间融资难题,严格落实金融支持实体经济发展的各项政策措施,引导金融机构创新适合民营企业、小微企业的融资模式,积极稳妥推进互联网金融、股权债权众筹等直接融资模式,拓展民间融资渠道。

(二)推进产业创新升级,构建晋中特色现代产业体系

坚持夯实一产、做强二产、加快三产的发展思路,着力构建农业基础比较稳固、装备制造业比较发达、第三产业发展迅速、吸纳就业能力不断强化、科技贡献份额不断提升、核心竞争力不断增强的现代产业体系。

聚焦提质增效,推进工业新型化。坚持以集群化、园区化、循环化、高端化为方向,加快工业化和信息化融合步伐,全面提升工业新型化水平。改造提升传统产业。突出延伸产业链、提升价值链,加大煤、焦、冶、电等行业技术改造力度,继续推进现代化矿井和安全高效矿井建设,发展先进产能,淘汰落后产能,提高原煤入洗率,增加煤炭附加值;坚持焦化并举、上下联产,建成灵石、介休两个焦化产业集聚区;积极承接山西科技创新城科研成果,围绕黑色煤炭绿色发展、高碳资源低碳发展,按照醇、苯、油发展线路,加快实施介休化工循环经济工业园区、阳煤昔阳化工园区等一批现代煤化工项目,积极推进榆社化工园区建设,打造山西中部新型煤化工基地;以东方希望、太钢万邦为龙头,重点发展氢氧化铝、氧化铝、电解铝和钢铁高端产品,加快建设灵石煤电铝一体化发展示范区;实施装机容量500万千瓦的5个低热值煤发电项目,全市电力装机容量由2015年的485万千瓦增加到1000万千瓦以上。做优做强特色产业。玻璃器皿产业要加快优化升级,提升行业市场竞争力,到2020年,销售收入由2015年的 23.4亿元增加到40亿元以上,建成全国玻璃器皿知名品牌创建示范区。玛钢铸造产业要加快推动太谷玛钢与平遥铸件融合发展,大力提升产品级次,销售收入由2015年的17.2 亿元增加到50亿元,建成全国玛钢铸造基地。纺机产业要重点发展智能化、高性能、高端纺机装备,加快壮大纺机产业集群,巩固我市“中国纺织机械名城”地位,销售收入由2015年的19.9亿元增加到50亿元以上。液压产业要发挥榆液品牌、技术和人才优势,重点建设太重榆液工业园,销售收入由2015年的 5.8亿元增加到80亿元,建成全国高性能液压元器件基地。碳素产业要以高技术含量、高附加值产品为重点,大力发展碳纤维、超高功率石墨电极等复合材料。医药产业要以生物制药为突破,以广誉远国药、德元堂药业为重点,加快推进晋中开发区、太谷和榆社三个医药工业园建设,形成以化学原料药及制剂、经典国药及现代中药为特色的医药工业体系,销售收入由2015年的21.9亿元增加到100亿元。食品产业要以平遥牛肉、祁县伊利、统一为龙头,重点推进畜禽、食醋、小杂粮等食品加工,销售收入由2015年的 71.2亿元增加到120亿元。培育壮大新兴产业。发展高端装备制造业,加快吉利甲醇、电动轿车投产和纯电动商用车项目建设,积极引进汽车零部件配套企业,构建新能源汽车产业链,全面推进新能源汽车装备制造园区建设。发展新材料产业,重点推进平遥煤化新型光学材料和石头环保新材料、灵石聚义宝鑫煤矸石制纤维、介休博创纯纳米氧化材料、晋中开发区中聚晶科蓝宝石晶体项目建设。发展清洁能源产业,抓好晋能2GW高效光伏电池组件项目,推进风能、光伏和生物质能发电,加快寿阳、和顺、昔阳等区域煤层气产业化开发利用,到2020年达到35-45亿立方米规模。发展节能环保产业,重点抓好山西国际能源脱硝催化剂等项目建设。发展信息技术产业,依托山西科技创新城,突出高端带动和集群发展,重点推进山西云智慧信息技术应用等项目建设。

转变农业发展方式,提升现代农业水平。壮大农业特色产业,按照“一核两区四带”区域发展布局,重点发展优质小杂粮50万亩,优质无公害设施蔬菜50万亩,优质水果100万亩,优质高产干果100万亩,中药材种植50万亩。促进一二三产融合发展,坚持粮经饲统筹、农林牧结合、种养加一体,促进全产业链循环化,重点推进“百企千场万户”养殖产业发展工程,建设标准化规模养殖场(小区)1000个;扶持壮大农产品加工企业,农产品加工业总产值年均增长20%;大力发展休闲农业与乡村旅游,休闲农业总收入突破100亿元。强化农业科技支撑,加大农民科技培训和农业技术推广力度,推进农产品标准化生产和质量安全绿色防控,“三品一标”认证率达到50%以上,主要农作物综合机械化水平达到80%以上。引深国家级现代农业示范区建设,重点实施好六县(区)“十个一”现代农业示范工程,提升农业创新发展能力。全面推进山西“农谷”建设,加速农业资源、资本、政策、人才、科技等各种要素的融合集成,用工业化方式来发展专业化基地农业、标准化品牌农业、工厂化制成品农业和多功能跨界农业,走出现代农业全产业链发展新路子。全市农业增加值年均增长10%,农民人均收入年均增长8.5%,到2020年率先进入基本实现农业现代化新阶段。

深化文旅融合发展,促进旅游业提档升级。着力构建大旅游发展格局,把文化旅游业作为战略性支柱产业培育,按照“一廊一弧一核两心三圈”全域旅游空间布局,全力做好“旅游业+”这篇大文章 ,探索“名胜景区+特色小镇+乡村旅游”发展模式,推动旅游业跨区域、跨城乡、跨产业融合,加快从景点景区旅游向全域旅游转变、单一门票经济向综合经济转变。着力完善旅游产品体系,以创建国家全域旅游示范区为统领,围绕构建晋商家国文化和太行山生态度假旅游两大产品体系,大力发展生态旅游、工业旅游、康养旅游、节庆旅游等新兴业态,建设30个特色文化旅游园区,推出100处无门票城郊休闲度假景观,创建30个3A级庄园景区或省级休闲旅游度假区,发展10个红色旅游经典景区。着力实施品牌提升工程,全市游客满意度指数继续保持山西首位,实现全市A级景区总量翻番、县县有4A景区,打好古城、大院、红色、民俗、山水、演艺六张城市形象特色牌,打响“晋商故里·家国晋中”全域旅游新品牌,加快建设国家优秀旅游目的地和以平遥古城为龙头的晋商国际旅游目的地。全市旅游总收入年均增长20%以上,2019年实现旅游接待游客1亿人次、总收入突破1000亿元。

加强规划引导,大力发展现代服务业。抓住市场倒逼经济转型机遇,推动各类服务业加速发育成长。以中鼎物流园区建设为重点,发展壮大现代物流业,打造中部地区现代物流枢纽。全面开发利用各类文化资源,突出文化与科技、体育、城建等互动融合,推动文化产业创新发展。大力引导需求、创造供给,加快推动金融服务、健康养老、体育休闲、电子商务等新兴服务业发展。以创建“宽带中国”示范城市为契机,大力实施“互联网+”行动计划,促进各种新兴业态加速发展。

(三)统筹城乡发展,加快推进新型城镇化

坚持把推进新型城镇化作为促进城乡统筹的根本途径,按照“一轴两区、一核两心”总体布局,传承晋商历史文脉,打造特色城市品牌,建设生态宜居美丽晋中,到2020年,全市城镇化率达到60%,中心城区达到80%。

加快市城区向区域性中心城市迈进。进一步优化布局,拓展城市规模。深入推进太原晋中一体化发展,加快两市基础设施互联互通、共建共享。按照“北进、西联、南扩、东延”的思路,以大学城、科创城、汽车城为重点,全面完善北部、西部、东部配套基础设施,加快南扩步伐,以兴办产业、改善设施、集聚人群为重点,实质性推动 “一市两区”建设,不断增强市城区集聚辐射能力。进一步完善设施,强化城市功能。坚持新区集中连片大开发、旧城改造整治大提升,全面实施公共服务、出行畅通、生态文明、设施提质、住房改善、城中村改造六大惠民工程, 28个在建的大型公建和商业综合体项目全部竣工运行,新实施东瑞创意街、冰雪世界等一批重大标志性工程,全面提升城市公共服务能力;开通晋中太原城际铁路、2条城市快速环路,改造提升安宁街、蕴华街、文苑街、新建路、中都路等10条主干道和43条小街巷,市民出行更加畅通快捷;抓好太榆排退水渠系改扩建项目和安宁街西延排水管网建设工程,推进综合管廊建设试点,加快“海绵城市”建设;每年新建6个便民服务市场、10个水冲式公厕,基本满足市民需求;合理规划布局,充分利用地上地下空间,每年新增1000个公共停车位、5000个小区停车位,到“十三五”末,公共停车位达到 1.3万个,有效解决市城区停车难问题;全面完成6大片区、19个棚户区、45个城中村改造任务,群众居住条件得到明显改善。进一步优化环境,提升城市品位。建成晋商公园三四期、雅乐公园和潇河湿地三期、学府生态湿地、东贾生态湿地公园,每年新建6个5000平方米以上公共绿地,实现300米见绿、500米见园。建立完善长效机制,扎实开展城市环境综合整治,突出抓好市城区街巷街景、小广告清理、车辆乱停乱放、私搭乱建、马路市场、小区环境卫生六大专项整治行动,切实解决城市脏乱差问题。加强城市文化建设,大力传承晋商文化,弘扬晋中精神,全面提升市民素质。到2020年,市城区人口达到90万人,建成区规模达到90平方公里,基本实现城市交通立体化、城市风貌都市化、居住环境生态化、功能设施现代化。

推进宜居县城、特色小镇和美丽乡村建设。全面推动县城扩容提质,加大市政基础设施和公共服务设施建设力度,加快新区建设和旧区改造,增强县城综合承载力和辐射带动力,引导生产要素、优势资源向县城集中,落实户籍制度改革政策,促进农村人口市民化,到2020年县城人口达到合理规模,支持具备行政区划调整条件的县撤县改市。围绕产业发展优、设施功能全、生态环境美、居住条件好、带动能力强目标,创建一批布局合理、特色鲜明的旅游小镇、现代农业小镇、新型工业小镇、传统手工业小镇、商贸小镇、生态园林小镇,以15个国家重点示范镇为带动,加快推进“五建设两整治”,将小城镇建设成为推动县域经济、辐射乡村发展的中心。按照家园美、田园美、生态美、生活美“四美”标准,打造200个高标准美丽乡村示范村,全面推进完善提质、农民安居、环境整治、宜居示范四大工程,不断改善农村人居环境,建立健全农村服务体系,加强国家级传统村落保护性开发,形成现代文明与乡村生态文明交相辉映的城乡发展新格局。

加大城乡基础设施建设力度。优化城乡交通条件,继续完善“六纵六横六循环”公路网,重点新建晋中-昔阳-衡水、龙城高速北延、祁县-离石三条高速公路,新增通车里程157公里;新建和改造一级公路565 公里、二级公路 233公里;完成农村公路网建设1500公里,实现县乡公路危桥全治理;每年推进2000公里“四好农村路”建设,到2019年提前一年达到“四好农村路”标准,实现二级以上客运站全覆盖。加强水利设施建设,重点抓好以东山供水、中部引黄为骨干的大水网工程、平川五县小水网工程和小水库更新建设工程,以东山灌区、汾河灌区、潇河灌区为龙头的高效节水灌溉工程,巩固提升农村饮水安全标准,解决50万人饮水安全问题。推进电网建设,深入实施农网升级改造和低电压治理,提升区域性电力供应保障能力。全面提升供气、供热、污水处理水平,到“十三五”末,全市新建改造燃气管网410公里,普及率达到96%以上;新增供热面积1000万平方米,普及率达到95%;城市污水处理率达到95%,垃圾无害化处理率达到95%以上。

全面加强城乡规划管理。发挥规划引领作用,推行多规合一,完成10个县市城市总体规划和近期建设规划修编、5个县县域乡村建设规划和智慧城市、综合交通、排水防涝、环卫设施等基础功能专项规划;加强规划执法,确保规划的严肃性和权威性。深化城市管理综合执法体制改革,建设一体化数字城管执法指挥平台,推进管理权限下放、属地职责下沉,调动街道、社区参与城市管理的积极性。建立全市域市容市貌综合管理考核评比机制,开展第三方考评和社会公众满意度测评,探索实行末位问责机制。

各位代表,城市的发展为了人民,城市的发展必须紧紧依靠人民。“十三五”期间,我们要以创建全国文明城市为引领,全面开展国家园林城市、国家卫生城市、国家环保模范城市“四城联创”。全市人民要发扬主人翁精神,积极投身创建活动,共建美丽城市,共享美好生活!

(四)发展壮大县域经济,夯实市域经济发展基础

县域经济是全市发展的基石,强县才能强市。要坚持市统筹、县为主,市县联动推动县域经济争先进位、创新突破、转型升级。

做强做大特色经济。落实主体功能区规划,划定生态保护红线、农业开发空间红线、城镇开发边界控制线,明确县域经济的功能定位和发展方向,加快榆次(含开发区)、介休、平遥3县(区、市)全面开发,统筹推进太谷、祁县、寿阳、昔阳4个农产品主产区和灵石、榆社、左权、和顺4个生态功能区的保护性开发,加快寿阳、昔阳、和顺、左权、榆社东山五县区域经济的发展,形成各有侧重、各具特色的县域经济发展格局。推进扩权强县、简政放权,探索在财政支持、融资、扩权、聚才、用地等方面进一步为县域经济发展“松绑”助力,增强县级统筹经济社会管理和基本公共服务供给能力。建立完善县域经济发展推进机制,确保全面挺进全省第一方阵各项指标圆满完成,并在全省县域经济发展中实现争先进位。

大力发展民营经济。抓住中央和省新一轮扶持民营经济发展的重要机遇,扎实开展“千名干部入企服务”和“各类项目大起底”活动,进一步完善落实扶持民营经济发展的政策措施,切实帮助企业解决运行和项目建设中的实际困难。加强分类指导,梯次推进,持续深入开展中小企业规范化管理提升行动,重视企业品牌建设,提升企业核心竞争力,力争到“十三五”末,规模以上民营企业普遍建立规范化管理体系。全面加强企业家队伍建设,实施企业家培训工程,推动我市民营经济重振雄风,再创辉煌。到“十三五”末,全市民营经济增加值比2010年翻一番。

加快发展园区经济。改革开发区管理体制,精干管理机构,转变管理职能,切实简政放权,增强内在活力,推进晋中经济技术开发区扩容提质,努力打造多功能综合性国家级经济技术开发区。进一步提升榆次工业园区、祁县开发区、介休开发区和灵石中煤循环经济园区基础设施水平,坚持有所为、有所不为,科学选择战略性新兴产业,整体规划、滚动开发,推进集聚集群和转型发展。统筹整合区域内开发区、产业园区和企业孵化器等平台,支持有条件的工业园区和产业园区创建省级经济技术开发区,加快把开发区打造成为人才高地、创新高地和产业高地。

(五)实施精准扶贫,坚决打赢脱贫攻坚战

以四个贫困县为主战场,在精准施策、精准推进、精准求效上下功夫,整体谋划、统筹实施,实现科学治贫、精准扶贫、根本脱贫。

精准实施六大攻坚工程。实施特色产业扶贫工程,大力发展特色种植、绿色养殖和光伏发电、乡村旅游等新型产业,支撑12.3万贫困人口增收脱贫;实施易地扶贫搬迁工程,完成1.9万贫困人口易地搬迁脱贫;实施生态补偿脱贫工程,帮助0.8万贫困人口稳定脱贫;实施教育培训就业工程,通过教育资助和就业培训促进1.7万贫困人口实现脱贫;实施政策保障兜底工程,确保2.89万失能贫困人口社保兜底脱贫;实施社会力量帮扶工程,凝聚全社会参与脱贫攻坚的强大合力。同时,要下大力气补齐贫困地区基础设施和公共服务两个短板,着力完善交通、水利、电力等基础设施,全面提升教育、医疗、科技、文化、信息等公共服务水平。

健全完善脱贫保障机制。强化财政扶贫投入增长机制,年度财政扶贫资金总量、增幅保持“双增长”;完善扶贫资源整合机制,支持贫困县统筹整合涉农资金和财政性扶贫资金捆绑使用;建立金融扶贫精准服务机制,争取更多长期低成本扶贫再贷款配额;探索资产收益扶贫机制,对部分财政资金投入项目形成的资产折股量化到贫困村和贫困户。

全面落实脱贫攻坚责任。严格落实各级党委、政府的主体责任,强化行业和部门的帮扶责任,充分发挥包村干部、驻村工作队、第一书记三支力量的作用,层层签订脱贫攻坚责任书,确保每项工作有人组织、有人负责、有人落实。建立资金项目监管责任制,实现任务、责任、资金、权力“四到县”,确保资金发挥最大效益。建立完善脱贫考核机制,按年度目标任务严格评估验收,建账销号,脱贫到人,对不能如期完成脱贫任务的严格问责。

各位代表,脱贫攻坚是全面建成小康社会的头等大事和第一民生工程。从今年开始,我们将每年完成脱贫4万人以上,确保2019年贫困县全部摘帽,确保2020年19.59万农村贫困人口全部脱贫!

(六)持续改善生态环境,推进绿色低碳发展

牢固树立绿水青山就是金山银山理念,把生态环境保护放在更加突出的位置,贯穿于经济社会发展全过程,着力发展绿色产业,筑牢绿色屏障,践行绿色生活,共建绿色家园。

加大环境治理力度。实行最严格的环境保护制度,以“控煤、治污、管车、降尘”为重点实施大气污染防治,严格控制燃煤污染,推进重点行业综合治理,加快淘汰黄标车和老旧车辆,严格管控扬尘污染。加强饮用水源地保护,实施汾河干流、漳河等流域生态修复,完成潇河市城区段治理,推进城镇污水处理提标改造,基本消除城市建成区黑臭水体。

推动低碳循环发展。落实循环发展引领计划,加快建立循环型产业体系,重点在电力、交通、建筑、冶金、化工、石化等行业推行循环型产业链和共生产业模式;推进生产、生活系统循环链接,积极开展循环经济重点领域试点示范创建工作。大力发展绿色交通,优化完善市区及各城镇的公交规划,扶持、鼓励公共自行车发展,中心城区公交站点500米全覆盖,具备条件的行政村全部通客车,到2020年市城区公交出行分担率达到23%以上、各县(区、市)18%,建设“公交都市”。高效节约利用资源能源,大幅降低煤基资源消耗,坚决完成焦化、电力、水泥重点行业淘汰落后和过剩产能任务,加快火电、冶金、化工、建材等高耗能行业节能技术改造,提升建筑能效水平,实行最严格的水资源管理制度和节约用地制度,完善生态环境有偿使用制度,推行合同能源管理和合同节水管理。

打造生态安全屏障。实施生态环境修复工程,加快太行、太岳两山生态修复,全面落实矿山生态环境恢复治理补偿机制和企业责任机制,推进采煤沉陷区、采空区、水土流失区、煤矸石山生态环境修复,到2020年基本解决采煤沉陷区受灾群众安居问题。加快创建省级林业生态市,坚持“山上治本、身边增绿、产业富民、林业增效”,构建“双百公里林果带、千里林业生态圈”林业发展格局,每年完成造林合格面积35万亩以上,林木绿化率平均每年增加1个百分点以上,森林覆盖率达到26%以上。

(七)全面推进改革创新,激活发展内生动力

将供给侧结构性改革与转型综改试验区建设结合起来,全面落实中央、省委全面深化改革任务,抓住重点领域和关键环节,着力破解制约经济社会发展的重大体制机制问题。

推进晋中108廊带示范区一体化改革。用足用好省政府九大专项扶持政策,以推动30项重大事项为载体,积极探索产业集聚、文旅融合、城镇发展、生态建设、交通体系、政策创新六个方面一体化发展路径;积极推进产业园区共建,大力发展“飞地经济”,形成跨县域调配资源、跨县域产城共建的工作经验;优先实施区域整体环评、土地管理创新等重大改革试点。

推进农村各项改革。2017年完成农村土地承包经营权确权登记颁证,同步建成县乡村三级联动的农村产权流转交易市场体系;深入推进宅基地、集体建设用地等农村产权改革,巩固农村林权、水权改革成果,加强农村“三资”管理;落实城乡建设用地增减挂钩政策,完成介休、太谷、祁县、榆次等国家和省改革试点任务。创新农业经营模式,推动土地有序流转,积极发展多种形式适度规模经营,培育壮大生态庄园、家庭农场、专业大户、农民合作社等新型农业经营主体。探索建立农业信贷担保体系,扩大农业保险覆盖范围,引导带动更多资金投向农业。

推进煤炭供给侧结构性改革和电力市场售电侧改革。探索组建区域性煤炭销售集团,建立市域内煤炭购销联合机制;建立“优胜劣汰”长效机制,减少无效供给;建立煤炭企业转型促进机制,鼓励发展非煤产业。落实电力市场售电侧改革要求,鼓励符合条件的企业和社会投资主体组建有增量配电业务的售电公司,在有条件的县市探索建设以园区为主的区域电网。

推进旅游开发管理体制改革。创新旅游综合管理体制,加快构建旅游管理执法“一盘棋”格局。理顺景区景点管理体制、经营机制,推行管理权、经营权分离,大力培育旅游业市场主体,进一步激发旅游市场活力。理顺利益分配关系,积极探索并加快形成景区共享发展的利益联结机制。引进国内外大集团,组建跨区域、跨行业、跨所有制的旅游集团公司,破解旅游资源整合、产品开发、市场融资等难题。

推进科技创新。深化科技管理改革,完善科技创新配套政策,加快形成推动创新创业的发展环境。大力发展科技孵化器、众创空间、公共技术研发平台,积极搭建公开统一的科技管理和服务平台,推动政产学研协同创新,做大做强一批高新技术企业和创新型企业,全面提升城市创新发展能力。积极促进“大学城”向“科学城”转型,建立市校联动常态化合作机制,促进高校科技资源与企业创新转型深度融合,努力打造区域科研创新高地。坚持人才培养和引进并举,完善人才评价激励政策和服务保障体系,全面加强科技人才队伍建设。

推进金融创新。创新机构供给,吸引各类金融机构入驻我市,支持晋中银行快速发展,推动农商行和村镇银行县域全覆盖,探索建立全功能金融服务集团。创新产品供给,扩大“助保贷”“税易贷”等金融产品覆盖面。发展直接融资,健全企业上市扶持培育机制,打造全市“四板”市场。创新融资机制,加大对种子期、初创期企业投资项目的金融支持力度,鼓励通过债权、股权、资产证券化等方式支持重大基础设施项目建设。建立金融稳定协调机制,有效防范区域性和系统性金融风险。

(八)全力保障和改善民生,提升人民群众的获得感和幸福感

发展是第一要务,民生是第一目标。越是经济下行压力大,越要高度重视民生。坚持从群众最关切的问题抓起,从群众最期盼的事情做起,每年办一批利民惠民实事,让发展成果更多、更公平地惠及全市人民。

全面加强就业和社会保障工作。实施更加积极的就业创业政策,重点抓好大学生、农村转移劳动力、城镇就业困难人员等群体的就业工作,鼓励以创业带动就业,不断提升就业创业服务水平。“十三五”期间,全市城镇新增就业14万人,城镇登记失业率控制在4.2%以内。建立更加公平可持续的社会保障制度,全面实施机关事业单位养老保险制度改革,统一城乡居民基本医疗保险制度,全面实施城乡居民大病保险制度,加强各项社会保险扩面征缴。健全城乡居民最低生活保障等社会救助体系,做好扶老、助残、救孤、济困工作。

积极推进全国养老服务业综合改革试点工作,建设以居家养老为基础、社区养老为依托、机构养老为补充的养老服务体系,到“十三五”末,每千名老年人拥有养老床位40张,养老服务设施覆盖80%的城市社区,农村日间照料中心覆盖千人以上行政村。完善住房保障体系,改造棚户区、城中村住房3.8万套和农村危房1.94万户。

统筹推进社会事业发展。加快建设现代化教育强市,继续推进学前教育三年行动计划,巩固提高九年义务制教育发展水平,率先通过市域义务教育基本均衡国家评估认定,实现义务教育优质均衡;推进高中学校标准化建设,2017年全部通过省级验收;落实职业教育行动计划,推动地方与驻地院校资源共享、深度合作;推进城乡教育一体化发展,全市新增城镇中小学和幼儿园50 所;全面改善薄弱学校基本办学条件,加快补齐农村教育短板。推进健康晋中建设,把人民健康放在优先发展的战略地位,抓好城市公立医院改革试点,健全分级诊疗体系,加快省级医院在我市项目的建设,完成市一院迁建和市妇幼保健院、传染病医院、三院、四院改扩建项目,加大妇幼基础设施建设和妇产儿科人才培养力度,保障生育政策调整的民生急需,到2020年,全市新农合参合率稳定在99.6%以上,优质医疗资源达到全省上游水平,二级以上医院全面推行网上预约诊疗服务,建立覆盖城乡居民的基本医疗卫生制度。加强基础能力建设,落实“四个最严”要求,严格食品药品监管,提高食药安全水平。深入实施全民健身国家战略,提升体育公共服务水平。完善公共文化服务体系,深入开展全民文化行动,重点推进国家级晋中文化生态保护实验区建设、市保以上文物古建抢救保护全覆盖、舞台艺术精品创作工程,到2018年,全面建成以“九化五机制”为标准的国家公共文化服务体系示范区。

毫不松懈抓好安全生产。按照“党政同责、一岗双责、失职追责”的要求,严格落实党委、政府领导责任和部门监管责任,强化企业主体责任。坚持安全风险分级管控和隐患排查治理,深入开展安全生产大检查和专项整治,从严实施“三个清单”监管和“四个一律”惩戒措施,进一步落实工商注册企业安全监管责任全覆盖,建立健全安全生产企业“黑名单”制度和信用挂钩联动机制,坚决执行安全生产考核“一票否决制”,努力减少一般事故,遏制较大事故,坚决防止重特大事故,促进全市安全生产形势持续稳定好转。

创新和加强社会治理。实施“七五”普法规划,加强社会治安综合治理,完善立体化社会治安防控体系,深化平安晋中建设。改进信访和人民调解工作,完善社会矛盾纠纷预防化解机制,深化风险隐患大排查大整治工作,加强应急管理和防灾减灾能力建设,做好舆情监控引导,维护社会和谐稳定。建立覆盖全社会的信用信息系统,加快建设“诚信晋中”。做好双拥和人防工作,加强国防后备力量建设,促进军民融合深度发展。

各位代表,今年是实施“十三五”规划的第一年,也是决胜全面小康的开局之年。全市上下认真贯彻落实中央、省一系列决策部署和政策措施,按照省委“一个指引、两手硬”的重大思路和要求,围绕市委确定的“苦干三年,全面挺进全省第一方阵”奋斗目标,强力推进十大攻坚战役,全市经济运行稳中有进、稳步向好,主要经济指标增幅居全省前列。现在距年底不到三个月时间,我们要坚定信心、乘势而上,进一步细化分解任务,层层压实责任,强化工作措施,确保圆满完成全年各项目标任务。

三、建设人民满意的有为政府

以加快政府职能转变为核心,推进治理体系和治理能力现代化,努力建设人民满意的法治政府、创新政府、廉洁政府和服务型政府。

强化法治思维,提升依法行政能力。牢固树立法治意识,自觉运用法治思维和法治方式履行职责,把政府工作全面纳入法治轨道。推行权力清单和责任清单制度,实施市场准入负面清单,加强对权力运行的监督和制约。严格执行公众参与、专家论证、风险评估、合法性审查和集体讨论决定的决策程序,提高依法决策水平。全面推行政府法律顾问制度,严格落实行政执法责任制,加强规范性文件管理和行政复议工作,提升依法行政水平。自觉接受人大及其常委会的法律监督和政协民主监督,切实加强审计监督,重视社会和舆论监督,让权力在阳光下运行。

强化为民意识,提升政务服务能力。以敬民之心,行简政之道。坚持放权、管理、服务、效能多管齐下,协同推进。加大政务公开力度,完善运行“市民之家”政务服务平台, 建设“互联网+政务服务”体系,大幅提升政务服务智慧化水平,营造审批事项少、行政效率高、行政成本低、行政过程公开透明的政务环境。

强化责任担当,提升工作落实能力。建立周碰头、月调度工作推进机制,实行定期向市委常委会报告工作和向人大、政协常委会通报情况制度,完善重点工作督查制度和考核奖惩制度,推动各项工作高效落实。建立完善容错纠错机制,推行干部任期目标责任考核,鼓励探索、宽容失误,以结果论成败、行奖罚,激发广大干部干事创业的热情和活力。各级领导干部要坚持以上率下,带头真抓实干,什么事情最需要办,就亲自去抓什么事情,什么问题最难办,就带头去解决什么问题,以过硬的作风创造经得起实践、历史和群众检验的业绩。

强化从严治政,提升廉洁履职能力。严守纪律规矩,时刻绷紧政治纪律和政治规矩这根弦。认真落实党风廉政建设主体责任,严格执行中央八项规定精神和国务院“约法三章”要求,坚决惩治腐败,持续狠刹“四风”,厉行勤俭节约,严控“三公”经费,努力实现干部清正、政府清廉、政治清明。

各位代表,使命催人奋进,奋斗铸就辉煌。让我们在省委、省政府和市委的正确领导下,在市人大、市政协的监督支持下,团结和依靠全市人民,抢抓机遇、开拓创新,夙夜在公、真抓实干,为实现全面挺进全省第一方阵、全面建成小康社会的宏伟目标而努力奋斗!
